# Intellectual Profiles of Clinic-Referred Preschoolers

**DOI:** 10.1177/08295735231154670

**Published:** 2023-02-22

**Authors:** Fannie Labelle, Marie-Julie Béliveau, Karine Jauvin, Marc-Antoine Akzam-Ouellette

**Affiliations:** 1 Psychology Department, Université de Montréal, Montréal, QC, Canada; 2 Hôpital en santé mentale Rivière-des-Prairies, CIUSSS - Du Nord-de-l’île-de-Montréal, Montréal, QC, Canada

**Keywords:** preschoolers, intelligence, comorbidity, psychiatry, diagnosis

## Abstract

Intellectual impairments in preschoolers have been widely studied. A regularity that emerges is that children’s intellectual impairments have an important impact on later adjustments in life. However, few studies have looked at the intellectual profiles of young psychiatric outpatients. This study aimed to describe the intelligence profile of preschoolers referred to psychiatry for various cognitive and behavioral problems in terms of verbal, nonverbal, and full-scale IQ and to examine their association with diagnoses. Three hundred four clinical records from young children aged under 7 years and 3 months who consulted at an outpatient psychiatric clinic and who had one intellectual assessment with a Wechsler Preschool and Primary Scale of Intelligence were reviewed. Verbal IQ (VIQ), Nonverbal IQ (NVIQ), and Full-scale IQ (FSIQ) were extracted. Hierarchical cluster analysis using Ward’s method was employed to organize data into groups. The children had, on average, a FSIQ of 81, which is significantly lower than that expected in the general population. Four clusters were identified by the hierarchical clusters analysis. Three were characterized by low, average, and high intellectual ability. The last cluster was characterized by a verbal deficit. Findings also revealed that children’s diagnoses were not related to any specific cluster, except for children with an intellectual disability with, as expected, low abilities. Children referred to an intellectual assessment in an early childhood mental health clinic showed an altered intellectual development, more specifically in the verbal domain.

## Introduction

A mental disorder is generally characterized by a disturbance in an individual’s cognition, emotional regulation, or behavior and is associated with distress or functional impairment (World Health Organization [WHO], 2022). Mental disorders include anxiety disorders, depression, bipolar disorder, post-traumatic stress disorder, schizophrenia, eating disorders, disruptive behavior and dissocial disorders, and neurodevelopmental disorders (ND; WHO, 2022). A recent meta-analysis of epidemiological studies (*N* = 18 282) showed that up to 20% of children under 7 years old were identified with mental disorders (Vasileva et al., 2021). Charach et al. (2020) found similar results among preschool children attending primary or community health services in their recent systematic review and meta-analysis, reporting that up to 18% were identified with a mental problem. Children attending mental health services clinics are likely to have various ND (Hansen et al., 2018). Moreover, children who consult for various ND such as language impairment, developmental coordination disorder, or autism spectrum disorder (ASD) frequently have lower intelligence (Courchesne et al., 2018; Cunningham et al., 2018; Gallinat et al., 2014).

Intelligence has been shown to be predictive of various life outcomes such as social maladjustment (Racz et al., 2017) and poorer educational achievement (Calvin et al., 2010; Deary et al., 2007). This is particularly true for children with multiple mental and neurological problems (Elbro et al., 2011). An intellectual profile is often recommended as an important part of the comprehensive assessment of a child (Reynolds et al., 2021). However, given that there is currently almost no knowledge of the intellectual characteristics of consulting children, professionals cannot know whether the results from their intellectual assessments are typical or not of this population. Moreover, knowing more about the intellectual profiles of children with mental disorders should be integrated into knowledge on the development and progression of mental disorders and will enable these children to be provided with appropriate services. The present study, therefore, aims to describe and investigate the intelligence profiles of clinic-referred preschoolers and examine their relationship to psychiatric diagnoses.

### Intelligence

Intelligence is essential to the adaptation of individuals (Gottfredson, 2007). Based on the Cattel-Horn-Carroll (CHC) model (McGrew, 1997), the *g* factor represents general intelligence. The *g* factor can also be divided into more specific subdomains (Schneider & McGrew, 2018). Among these subdomains, fluid intelligence (Gf) is described as the ability to solve new problems regardless of acquired knowledge. Crystallized intelligence (Gc) is characterized by knowledge acquired through experience, whether culture or language (Schneider & McGrew, 2018). Visuospatial processing (Gv) is the ability to perceive and manipulate nonverbal images for solving problems and the ability to use mental imagery to perform spatial reasoning (Schneider & McGrew, 2018). Both Gf and Gv are measures of nonverbal intelligence. This model is increasingly used to conceptualize intelligence in intellectual instruments (Kranzler et al., 2016). The g factor is then reflected by the intellectual quotient (IQ). In clinical settings, intelligence tests are routinely administered to children as part of their psychological assessment (Kranzler et al., 2016). The most frequently administered intelligence tests are the Wechsler scales (Kranzler et al., 2016). Knowing that cognitive ability is a broad construct with various domains, each of them can be selectively impaired or intact, especially among clinical populations, therefore rendering more essential intellectual assessments of consulting children.

### Intelligence in Clinical Populations

The assessment of intelligence is standard practice in psychology and neuropsychology. In addition to identifying children with difficulties, it allows for assessing a patient’s suitability for psychological interventions (Reynolds et al., 2021). However, there is almost no scientific knowledge about the intellectual profiles of preschool children consulting in psychiatry. Thus, not only is there a risk of not taking this factor into account in service provision, but it is also not known whether the profiles revealed through clinical assessments are typical or not among this population. To our knowledge, only one study has described the intelligence of preschoolers consulting in psychiatry. Koushik et al. (2007) studied 108 children aged between 3 and 7 years old referred to a day-treatment program for a variety of cognitive and behavioral problems. Children were then grouped into five subtypes according to their cognitive pattern highlighting the high variability of the cognitive capacities among children consulting in psychiatry. For three of these groups, the difference was in their global IQ (Low, average or high ability). The two other groups, each representing a quarter of the sample, differed in verbal abilities relative to nonverbal abilities. Some had a profile characterized by a verbal deficit while others had a nonverbal deficit. Lower verbal abilities were more associated with diagnoses of language disorders and lower nonverbal abilities were more associated with diagnoses of ADHD and externalized disorders (Koushik et al., 2007). These results show the importance to describe all the specific cognitive abilities of children in clinical settings since the presence of some deficits can lead to the exploration of new clinical hypotheses. However, a replication of this study is warranted. In another study with referred children with developmental delays aged between 5 and 7, results showed that nonverbal IQ was higher than verbal IQ for all children, even for those with normal language development (Liao et al., 2015). Authors concluded that a discrepancy between verbal and nonverbal IQ is common in children with developmental disorders (Liao et al., 2015).

Validation studies of intellectual tests have been conducted with clinical populations to inform on the clinical utility of intellectual assessment tools, suggesting that a variety of intellectual profiles can be identified and variation among intellectual profiles can be related to child disorder (Weiss et al., 2015). For example, children with language impairment had an intellectual profile characterized by poor performance on verbal scales while children with intellectual disability had poor performance on all scales. Conversely, children with giftedness had a profile characterized by high performance on all scales (Wechsler, 2004). However, to be included in the validation studies, children must not have any other comorbid conditions (Wechsler, 2004, 2013). Therefore, the results of these validation studies with clinical populations cannot be applied to children with multiple comorbid conditions that would be representative of clinical settings. In scientific and clinical domains, there is a trend to make categorical diagnoses and subsequent research includes only one or two of these diagnoses. In doing so, it alters the ecological validity of these studies as this does not reflect the clinical reality as most children referred for mental health services have high rates of comorbidity (Hansen et al., 2018). It is therefore important to study children with multiple diagnoses. In addition, the sample sizes of the validation studies are very small (<50 children) and therefore less generalizable to consulting children (Wechsler, 2004).

Only a few studies considered children with comorbidities, but these were with specific diagnoses (Language impairment: Elbro et al., 2011; ADHD: Katusic et al., 2011; Skogan et al., 2014; Takeda et al., 2012; Waschbusch, 2002). Thus, again, the results of these studies cannot be generalized to clinic-referred children whose problems are likely to be more severe and varied. Failure to identify children’s comorbidities is a very common limitation among studies while research on mental disorders in children under 7 years of age is already considered to be a neglected area (Vasileva et al., 2021). A better understanding of the overall diagnostic profile of children in relation to their intellectual profiles will shed new light on the association between intelligence and psychopathology among young children.

## Objectives and Hypotheses

The aim of this study is to describe and investigate the intelligence profile of preschoolers referred to psychiatry in terms of verbal, nonverbal, and full-scale IQ. Similar profiles to those found in the study of Koushik et al. (2007) are expected. In an exploratory way, a second objective is to examine the relationship between intelligence profiles and psychiatric diagnoses. Given the exploratory aspect of this objective, no hypotheses are made except for the obvious expectation that diagnoses of intellectual disability will be associated with low intelligence.

## Method

### Participants

Participants are preschoolers from a large metropolitan area who consulted at an outpatient psychiatric clinic specialized for children under six. This clinic offered services for any problem, not primarily related to an ASD diagnosis, presented by a child for which a physician wished to obtain a psychiatric opinion. Data were extracted from the information available in the medical records. The ethical and administrative hospital authorities authorized access to the clinical records of all 931 children assessed between 2000 and 2016. Medical files were reviewed by research assistants to extract their psychiatric diagnoses, intellectual assessments, as well as their personal and familial characteristics. Children must have had one intellectual assessment with a Wechsler Preschool and Primary Scale of Intelligence (WPPSI) for which the results are accessible and be aged under 7 years and 3 months. The final sample is composed of 304 participants.

T-tests and chi-squares were conducted to compare whether the subsample selected for this study differs from children who were not included in this subsample on socio-demographic variables. Results show that there is no statistical difference between the subsample and the large sample on mother and father education, mother and father country of birth, and child sex (*ps* ≥ .13).

### Psychiatric Diagnosis

Children first met a psychiatrist accompanied by a mental health specialist who provided first clinical impressions, diagnoses, and orientations to further services, which could include an intellectual assessment. Afterward, the child was seen again by the psychiatrist to review the diagnoses and recommendations. All diagnoses (at the first and second assessment) made by the psychiatrist were coded according to the DSM-IV-TR and ICD-10 by two independent blind judges. To avoid confusion, the current terminology used corresponds to those of the DSM-5-TR (American Psychiatric Association [APA], 2022) and consists of: (a) neurodevelopmental disorders (including communication disorders, ADHD, and motor disorders); (b) psychiatric disorders (including disruptive, impulse-control and conduct disorders, anxiety disorders, depressive disorders, and other relational problems) and (c) intellectual Developmental Disorder (IDD) and autism spectrum disorder (ASD). Although referrals for ASD (formerly called a pervasive developmental disorder) were not directed to this clinic, some children, after a thorough assessment, were diagnosed with previously unsuspected ASD. In addition, although intellectual disability is considered a neurodevelopmental disorder (APA, 2022), it has been considered separate from other ND since this diagnosis includes, among other criteria, an intellectual functioning deficit. Final diagnoses either established at the second or first psychiatric assessment were retained for the present study.

### Intelligence

Intellectual assessments were conducted in a clinical context by a psychologist or a neuropsychologist. Clinicians selected intellectual measures based on several factors such as age, child limitations, and availability of a Canadian or French version. Hence, intellectual tests were not randomly assigned to children. The intellectual assessment closest to the final psychiatric assessment was retained for the present study. Most of the children (*n* = 263) were assessed with the WPPSI-III (Wechsler, 2004). The previous (WPPSI—Revised Form; Wechsler, 1989) (*n* = 27) or next version (WPPSI-IV; Wechsler, 2013) (*n* = 14) were also administered, depending on the timing of the assessment. Three IQs were collected: Verbal IQ (VIQ), Performance IQ (PIQ, or nonverbal IQ (NVIQ)), and Full-scale IQ (FSIQ) (see Table 1). A total of 235 children have valid scores on all 3 IQs.

**Table 1.** Number of Children With an Available Score.

	FSIQ	VIQ	NVIQ
*n*	241	246	256
Missing	63	58	48
Total	304	304	304

*Note.* FSIQ = full-scale IQ; VIQ = verbal IQ; NVIQ = nonverbal IQ.

### Data Analyses

First, descriptive statistics were conducted. Correlational analyses were carried out between IQ scores and socio-demographic variables. Although the present study did not have data from an age-matched group of typically developing (TD) children, the use of standard scores based on normative data from the Wechsler scales allowed a comparison between referred and TD children. *T*-tests were calculated to compare children’s FSIQ, VIQ, and NVIQ to the one expected in the general population. Next, to determine the intelligence profiles, hierarchical cluster analysis using Ward’s method with squared Euclidean distance as a measure of similarity was employed. For this analysis, only children with scores on all three IQs are retained. Then, to examine the relationship between intelligence profiles and diagnostic categories, crosstabs were generated. Statistical analyses were conducted using SPSS statistics 25.0 (IBM Corp., Armonk, NY, USA).

## Results

### Participant Characteristics

Sample demographics are presented in Table 2. The final sample consisted of 304 participants (24 to 87 months; *M* = 57.75 months, *SD* = 12.08; 230 boys). Among the available date, only 46% of children were reported as French unilingual. Fifty-four percent of them also heard another language at home. After French, the most common languages in bilingual families were English (29%), Caribbean Creole (29%), Spanish (14%), and Arabic or Berber (12%). Over 80% of children were diagnosed with a neurodevelopmental disorder, more than 50% were diagnosed with a psychiatric disorder and <15% were diagnosed with an IDD or ASD. However, these groups are not independent. A child diagnosed with a neurodevelopmental disorder may also have been diagnosed with a psychiatric disorder. To obtain independent groups, children were further organized into subtypes. Five children received no diagnoses either at the first or second psychiatric evaluation but were retained for the cluster analysis. Thus, of 299 children with at least one diagnosis, a total of 105 (35.12%) children had a neurodevelopmental disorder only, 21 (7.02%) had a psychiatric disorder only, 135 (45.15%) children had both a neurodevelopmental and psychiatric disorder. Finally, all children diagnosed with IDD-ASD were grouped into a separate category (*n* = 38; 12.71%) regardless of the presence of other difficulties.

**Table 2.** Sample Demographics (*N* = 304).

Socio-demographic variables	*n* (%)
Sex	
Female	74 (24.3)
Male	230 (75.7)
Age (months)	
<36	4 (1.3)
36 to <48	68 (22.4)
48 to <60	99 (32.6)
60 to <72	84 (27.6)
72 to <84	47 (15.5)
84 to 87	2 (0.7)
Maternal education	
Elementary school or less	7 (2.3)
High school	134 (44.1)
DEC, DEP or equivalent	77 (25.3)
University	73 (24.0)
Missing	13 (4.3)
Mother’s country of birth	
Canada	165 (54.3)
Outside Canada	126 (41.4)
Missing	13 (4.3)
Father’s country of birth	
Canada	130 (42.8)
Outside Canada	117 (38.5)
Missing	57 (18.8)
Languages spoken at home	
French only	86 (28.3)
French and/or other(s)	100 (32.9)
Missing	118 (38.8)

*Note.* DEC (diplôme d’études collégiales) is a diploma of college studies. DEP (diplôme d’études professionnelles) is a diploma of vocational studies.

Correlations between socio-demographic variables and IQs are presented in Table 3. Children whose parents were born in Canada have higher IQs (*p* < .001). Also, increased maternal education is associated with a higher IQ score (*p* < .05). All IQs are strongly correlated to each other (*p* < .01).

**Table 3.** Correlational Analyses Between Socio-demographic Variables and IQ Scores.

	*n*	1	2	3	4	5	6	7	8
1. Mother’s country of birth	291	—							
2. Father’s country of birth	247	.72**	—						
3. Child’s age	304	.06	−.00	—					
4. Child’s sex	304	.15*	.12	.06	—				
5. Maternal education	291	.03	−.01	−.07	−.02	—			
6. VIQ	246	−.37**	−.43**	−.04	−.10	.23**	—		
7. PIQ	256	−.28**	−.31**	.10	−.01	.17*	.65**	—	
8. FSIQ	241	−.34**	−.37**	.05	−.06	.21**	.89**	.91**	—

*Note.* FSIQ = full-scale IQ; VIQ = verbal IQ; NVIQ = nonverbal IQ.

* *p* < .05. **p < .01.

### Intelligence Quotients

FSIQ (*n* = 241) varied from 40 to 128 (*M* = 81.18, *SD* = 17.82). VIQ (*n* = 246) varied from 45 to 126 (*M* = 76.32, *SD* = 16.67). NVIQ (*n* = 256) ranged from 45 to 137 (*M* = 90.63, *SD* = 18.78). FSIQ, VIQ, and NVIQ were all statistically inferior to the one expected in the general population (*t* [240] = −16.40, *p* < .001, *d* = −1.06; *t* [245] = −22.29, *p* < .001, *d* = −1.42; *t* [255] = −7.98, *p* < .001, *d* = −0.50). Finally, a paired *t*-test revealed the mean VIQ to be statistically inferior to the mean NVIQ for the whole sample (*t* [244] = −15.56, *p* < .001, *d* = −0.99). FSIQ, VIQ, and NVIQ were then observed for each of the diagnostic subtypes. For all subtypes, the mean VIQ is lower than the NVIQ (up to a 17-point IQ difference). As could be expected, the only subtype with all mean IQs below 2 SD is associated with being diagnosed with an IDD or ASD (see Table 4).

**Table 4.** IQ According to Diagnostic Subtypes.

	FSIQ		VIQ		NVIQ	
Diagnostic subtypes	*n*	*M* (*SD*)	*n*	*M* (*SD*)	*n*	*M* (*SD*)
Neurodevelopmental disorders only	83	79.43 (16.33)	83	74.40 (15.29)	89	89.80 (17.11)
Psychiatric disorders only	19	98.00 (14.78)	18	94.89 (11.88)	18	104.11 (15.24)
Neurodevelopmental and psychiatric disorders	115	82.83 (16.84)	119	76.86 (16.64)	123	93.33 (17.39)
IDD-ASD and other disorders	20	62.70 (16.08)	22	64.89 (13.72)	23	68.52 (18.40)

*Note.* FSIQ = full-scale IQ; VIQ = verbal IQ; NVIQ = nonverbal IQ; IDD = intellectual developmental disorder; ASD = autism spectrum disorder; M = mean; SD = standard deviation.

### Intellectual Profiles

According to the dendrogram analysis, the four-cluster solution provided the best fit the (*n* = 235). The first cluster is defined as an average nonverbal ability with verbal deficit (AVD): VIQ is 1 standard deviation (SD) below the mean and NVIQ is average (*n* = 103). The second cluster is characterized by low abilities (LOW) in all aspects with about 2 SD below the mean (*n* = 65). The third cluster is represented by average abilities (AVG; *n* = 37). Finally, the fourth cluster is characterized by high average abilities on all IQs (HAVG; *n* = 30; see Table 5).

**Table 5.** Prevalence, Gender Ratio, and Mean IQs for Each Cluster.

Cluster	Prevalence, *n* (%)	Gender ratio (boys [%])	VIQ	NVIQ	FSIQ
AVD	103 (43.83)	72 (69.90)	76.27	90.74	80.37
LOW	65 (27.66)	52 (80)	58.86	71.54	60.49
AVG	37 (15.74)	28 (75.68)	86.27	110.81	97.78
HAVG	30 (12.77)	22 (73.33)	106.43	113.77	110.17

*Note.* AVD = average nonverbal ability with verbal deficit; LOW = low abilities in all IQs; AVG = average abilities in all IQs; HAVG = high average abilities on all IQs; FSIQ = full-scale IQ; VIQ = verbal IQ; NVIQ = nonverbal IQ.

**Figure 1.**
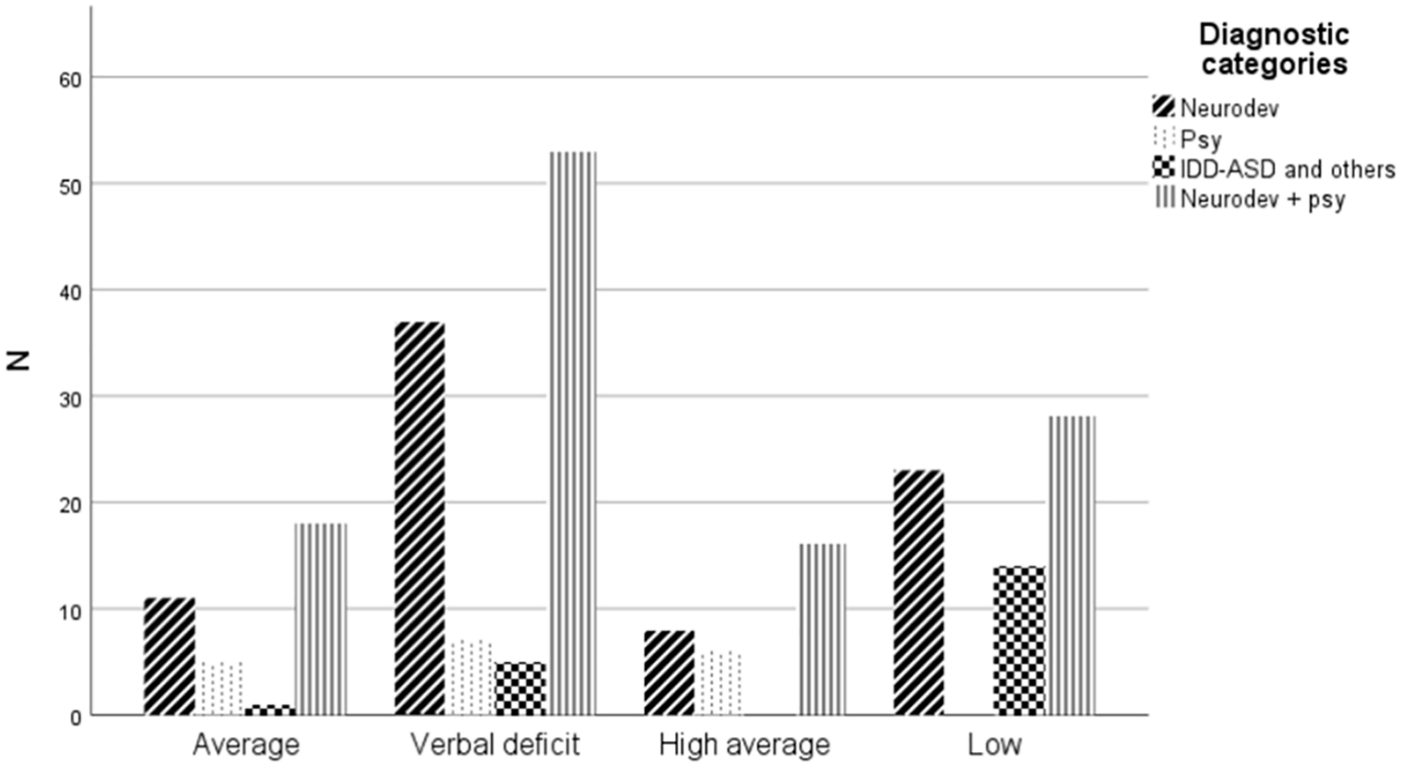
Crosstab between diagnostic subgroups and clusters.

A crosstab was generated. All the different diagnostic subgroups are represented in each cluster (AVG, AVD, HAVG, LOW) except for IDD-ASD. As expected, a large majority of children diagnosed with IDD-ASD are found in the low intellectual profile while no child with this diagnosis is in the HAVG group (see Figure 1). Children diagnosed with a ND with or without a psychiatric disorder are found in all clusters. Finally, no child with a psychiatric disorder only is in the LOW cluster.

## Discussion

### Intelligence Among Consulting Children

The aim of this study was to document the intelligence of preschoolers referred to psychiatry for various developmental and behavioral problems. As expected, results revealed that children referred to an intellectual assessment in an early childhood mental health clinic showed an altered intellectual development. Few children were diagnosed with an intellectual disability (<15%) and yet the average of the three IQs remains lower than expected for the overall sample. The IQs are normally distributed but the curve is shifted to the left, where the children’s mean intelligence is significantly lower than expected. However, the *SD* found in each distribution is comparable to the one found in a normal population (about 15 points). Verbal and nonverbal IQ means are low, but the nonverbal IQ is closer to the one expected in the general population and within the average range, albeit in the lower part of the average interval. Furthermore, the VIQ is statistically lower than the NVIQ (76 vs. 90) for the whole sample. Therefore, for a large proportion of clinic-referred children, their nonverbal abilities are higher than their verbal abilities. These results converge with several studies that show that children who consult for emotional and behavioral problems have language impairments (Benner, 2005; Benner et al., 2002; Hollo et al., 2014). Presumably, these language difficulties are reflected in their intellectual assessment. The significant difference found between VIQ and NVIQ was present at the full sample level but also for all diagnostic subtypes, and not specifically for children diagnosed with a language disorder. Validation studies of the WPPSI-III also reported discrepancies between VIQ and NVIQ in clinical groups (Wechsler, 2004). For example, children with mixed receptive-expressive language disorder (*n* = 27) have a difference of 2 IQ points in favor of NVIQ. The largest discrepancy found was for children diagnosed with ASD (*n* = 21) (18-point difference in favor of NVIQ). Children diagnosed with motor impairment (*n* = 16) also showed a difference between their NVIQ and VIQ of 15 points, in favor of the latter, while a difference of 4 points was observed for children with ADHD (*n* = 41) in favor of NVIQ. In the present study, a difference of 10 points is observed for the group of children with psychiatric disorders only. Much larger discrepancies between VIQ and NVIQ are reported in the present study than in the WPPSI-III validation studies. The complexity of the difficulties encountered by children referred to psychiatric clinics may partly explain these larger differences compared to those found in the validation studies where all children with comorbidities are excluded. These results support the idea that studies with larger clinical samples are needed and studies with consulting children in order to have better ecological validity. In sum, regardless of the diagnosis, children who consult in a psychiatric clinic have lower intelligence, particularly in the verbal domain.

The results obtained from the hierarchical clusters analysis showed four distinct profiles indicating high variability in the intellectual abilities of clinic-referred preschoolers. The four-cluster solution is similar to the cluster solution identified by Koushik et al. (2007) except that there is no cluster characterized by a nonverbal deficit. Indeed, in all groups, the verbal IQ is below the nonverbal IQ.

### Association Between Clusters and Diagnostic Subtypes

The association between intelligence profiles and diagnoses was examined in an exploratory way. As expected, children with a previous diagnosis of IDD-ASD were in the low intellectual ability profile. Also, no children diagnosed with a psychiatric disorder only were in the cluster characterized by low general intelligence. This suggests that children with a psychiatric disorder tend to have a more preserved IQ than children diagnosed with neurodevelopmental disorders. Otherwise, children with a ND only or with a psychiatric disorder were also found in the four intellectual profiles obtained from the cluster analysis. The cluster characterized by high-average intelligence is underrepresented among this clinical sample, representing 12.8% of all assessed children whereas this profile should be more common based on what is known about the general population. There were no distinct intellectual profiles according to diagnostic subtypes, except for the IDD-ASD group and therefore it is important to continue intellectual assessments as it is not possible to determine the intellectual profile according to the type of diagnosis received.

### Parental Characteristics

Maternal education was positively associated with the three IQs included in this study, which converges with the well-established literature about maternal education as a strong predictor of child IQ (Bornstein et al., 2013).

A large proportion of the children seen at this clinic are children of parents born outside Canada. Country of birth was available for 291 mothers and 247 fathers. Among them, just over half of the mothers (56.7%) and fathers (52.6%) were born outside Canada. These percentages are relatively similar to those found in the clinic’s metropolitan area (Statistics Canada, 2019). Children whose parents were born outside of Canada tend to have lower IQ scores. Even if heredity accounts for a large variation in intelligence, cognitive development can be influenced by environmental factors. Differences in mean IQs between cultural groups are well known (Weiss et al., 2015) and various adaptations of the Wechsler scales have been made across countries to take these differences into account. However, these differences in IQ according to the parents’ culture would be better explained by other mediating factors. Culture could be a proxy variable reflecting social and environmental inequalities (such as SES) that facilitate or hinder the cognitive development of young people (Weiss & Saklofske, 2020). Indeed, multiple factors may affect the association between maternal country of birth and intelligence. Moreover, it may be that children of immigrants are more likely to seek psychiatric care, hence the large proportion found in this study. It would be important to conduct further studies on this topic.

### Relevance to the Practice of School Psychology

It is recommended that the integration of young children with clinical-level behavioral and developmental difficulties into regular education settings be promoted (Oh-Young & Filler, 2015). The benefits of integration were supported by a recent meta-analysis of 24 studies with participants aged 3 to 21 years. The latter supports the positive effects of integration on academic performance and social interactions (Oh-Young & Filler, 2015). In addition, children with developmental disabilities benefit from inclusion in regular classes by interacting with typically developing peers (see Webster & Carter, 2007 for a report). Also, by identifying children with IDD, programs could be offered to these children in school settings to promote their emotional regulation and social behaviors (Jacobs et al., 2020). The choice of whether to place the child in a regular or special education class should be determined by the needs of the individual child. If the child's needs are not being met, the appropriateness of the placement should be questioned. In that context, the assessment of intelligence, particularly in children with special needs referred to psychiatric consultation, is crucial given the variability and heterogeneity of the intellectual abilities found in this study. A good understanding of the child's intellectual profile allows for referral to appropriate services based on the child’s cognitive strengths and weaknesses (Reynolds et al., 2021). Results of the current study showed that even when the consulting child has a lower-than-average nonverbal IQ than expected in the general population, this domain remains a personal strength in most cases. Given the verbal difficulties of referred preschool children, preference should be given to interventions that are not language-based. Educators could therefore be made aware that nonverbal skills, which are more preserved, should be more systematically solicited to support children’s new learning.

### Limitations and Future Research

The current study has several limits resulting from data extraction from clinical records. Intellectual assessments were not systematically provided and might have been provided to children suspected of intellectual deficits by the clinical team. Therefore, they would not be representative of all referred children. The representativeness of results was verified by comparing the socio-demographic characteristics of participants with and without an intellectual assessment. The children included in the present study did not differ significantly from all children consulting in this clinic regarding gender, mother’s and father’s education, and mother’s and father’s country of birth. Differences in the instruments used to assess intellectual abilities may also have affected the results. The subtests that make up the VIQ and NVQ scales are different depending on the version of the WPPSI. However, recent results showed that the different constructs measured by the Wechsler scales are generally the same and consistent across versions and revisions (Niileksela & Reynolds, 2019). Although the non-verbal measures of the Wechsler scales minimize the expressive demands of children, they are not language-free. In future studies, it would be interesting to see if the same association is found with nonverbal measures, for example, the Leiter international performance scale third edition (Roid et al., 2013) which is completely non-verbal. It is also important to raise the limitations of a retrospective study. At the time, the diagnoses made by psychiatrists were based on the DSM IV-TR and ICD-10 criteria. The assessment process was done according to the best practices of the time. However, revisions of these manuals have since been published. Some diagnostic criteria may have been added, removed or modified. For example, severity of IDD is now rated according to adaptative functioning. However, it is important to note that despite this change, no diagnosis of IDD was made at the time based solely on IQ.

Despite these limitations, one of the greatest strengths of the current study is the ecological validity due to the clinical sample used. It properly documents all diagnoses presented by patients, whereas most clinical studies consider only children with one specific disorder. Therefore, it better reflects the complexity of this clinical population. Also, the sample size of this study is much larger than the studies that had previously reported on children’s intellectual profiles including results from unpublished validation studies of intellectual tests.

Further studies should include children with heterogeneous difficulties to better represent clinical populations. Furthermore, longitudinal studies would allow for a better understanding of how intelligence, psychiatric disorders, and symptoms influence each other over the life course. Moreover, it would be important that future studies include a control group with TD children in which they are matched on socio-demographic variables to the children from the clinical population. In doing so, this would allow verifying if presenting difficulties great enough to require a psychiatric consultation is associated with lower intellectual capacities. Such studies are important to better understand the links between intelligence development and psychopathology, as well as, more specifically, why it is so common for the verbal sphere to be affected. Is this a characteristic of the children who consult? An indicator of the severity of their deficits? These are questions to be examined in future studies with other types of designs such as longitudinal designs or case-control studies. The integrative and wider perspective of the present paper allowed to demonstrate that a significant portion of consulting preschoolers will have intellectual delays, especially in the verbal sphere, and heterogeneous profiles.

## Conclusion

A small proportion of children affected with psychiatric disorders are referred for mental health services or receive treatment (Egger & Angold, 2006). For those who are referred, the main reason for psychiatric consultation during preschool age concerns problematic behaviors (Finello, 2011) but as seen, other impairments are often present, including an altered intellectual development. Children who need to consult a psychiatric clinic at a young age often have considerable developmental delays. A large proportion of preschoolers in need of child psychiatric services show lower IQs than the general population, in all IQ domains, but most markedly in the verbal domain. This is consistent with the large proportion who presented neurodevelopmental disorders, even if this psychiatric clinic was not aimed at assisting children with neurodevelopmental problems. Decision-makers and clinicians must be aware of these characteristics in order to allow earlier intervention when brain plasticity is greater and the long-term impacts of impaired cognitive abilities on their academic and social development are easier to prevent. As recommended by Rutter and Stevenson (2008), services must be based on the individual’s needs rather than on diagnosis. It is therefore essential to take intellectual development into account when offering services for children and not only focus on behavioral and emotional problems.
